# CagA-producing *Helicobacter pylori* and increased risk of gastric cancer: a nested case–control study in Korea

**DOI:** 10.1038/sj.bjc.6603309

**Published:** 2006-08-08

**Authors:** J Gwack, A Shin, C-S Kim, K-P Ko, Y Kim, J K Jun, J Bae, S K Park, Y-C Hong, D Kang, S-H Chang, H-R Shin, K-Y Yoo

**Affiliations:** 1 Department of Preventive Medicine, Seoul National University College of Medicine, 28 Yongon-dong, Chongno-gu, Seoul, 110-799, Korea; 2 Center for Health Services Research, Vanderbilt University Medical Center, Nashville, TN, USA; 3 Department of Preventive Medicine, Konkuk University College of Medicine, 322 Danwol-dong, Chungju-si, Chungcheongbuk-do 380-701, Korea; 4 Research Institute for National Cancer Control and Evaluation, National Cancer Center, 809 Madu1-dong, Ilsandong-gu, Goyang-si, Gyeonggi-do 410-769, Korea; 5 National Cancer Center, 809 Madu1-dong, Ilsandong-gu, Goyang-si, Gyeonggi-do 410-769, Korea

**Keywords:** gastric cancer, *Helicobacter pylori*, CagA, Cohort study, Korea

## Abstract

In a nested-case control study of 100 cases of gastric cancer and 400 matched controls in relation to virulence factors of *Helicobacter pylori* in a Korean cohort, CagA seropositivity was significantly associated with a higher risk of gastric cancer among *H. pylori*-infected subjects (OR=3.57, 95% CI 1.05–12.14).

Gastric cancer is the first major incident cancer with an age-standardized incidence rate of 69.6 in males and 26.8 in females per 100 000 in Korea, the highest in the world (Ferlay *et al*, 2004; Shin *et al*, 2005b). *Helicobacter pylori* (HP) was classified as a group I human carcinogen for gastric cancer by the International Agency for Research on Cancer in 1994 (IARC, 1994). However, despite the evidence that HP infection increases gastric cancer risk, the prevalence of HP infection does not always correlate positively with risk (Uemura *et al*, 2001; Peek and Blaser, 2002; Lunet and Barros, 2003). In fact, certain Asian and African countries with a high prevalence of HP infection have a low incidence of gastric cancer (Lunet and Barros, 2003).

One explanation for the above differences concerns virulence factors, such as cytotoxin-associated antigen (CagA) and vacuolating cytotoxin (VacA), produced by HP strains, that may be more carcinogenic to the gastric epithelium (Peek and Blaser, 2002). These factors can invade epithelial cells in stomach walls and induce epithelial responses with carcinogenic potential (Peek and Crabtree, 2006).

We previously reported a null association between HP infection and gastric cancer in a nested case–control study within the Korean Multi-Center Cancer Cohort (KMCC) (Shin *et al*, 2005a). We have therefore investigated the virulence factors, CagA and/or VacA seropositivity, in relation to gastric cancer susceptibility.

## MATERIALS AND METHODS

The Korean Multi-Center Cancer Cohort (KMCC) is a prospective cancer cohort based on four urban or rural areas in Korea (Yoo *et al*, 2002). Participants over age 30 years were recruited from 1993 through 2004. A detailed standardized questionnaire on general lifestyle, physical activity, dietary habit, reproductive factors, and past medical history was completed for each subject by interviewers at the time of recruitment. Blood and urine samples were donated voluntarily. Blood samples were then stored at −70°C and urine samples at −20°C. The study protocol was approved by the Institutional Review Boards of the Seoul National University Hospital and the National Cancer Center of Korea. All subjects provided written informed consent.

As of December 2002, 136 gastric cancer cases were identified among the 14,440 cohort members through a computerized record linkage to the Korea Central Cancer Registry database and the National Health Insurance database. Of these, we excluded gastric cancer cases diagnosed before recruitment (*n*=36). To validate a diagnosis of gastric cancer and to obtain additional detailed clinical information such as tumour site, a medical record review was undertaken in all such cases. For comparison, four controls from the eligible cancer-free cohort were matched to each cancer case by incidence density sampling based on age (within 5 years), gender, area of residence and the year of recruitment.

Sera were assayed using immunoblot kits (Helico Blot 2.1™, MP Biomedicals Asia Pacific, Singapore) to identify IgG antibodies specific for HP according to the manufacturer's instruction. CagA and VacA seropositivity and HP infection status were determined using these kits. Sensitivities for HP infection, and CagA and VacA seropositivities have been reported to be 99, 99 and 93%, and specificities to be 98, 90 and 88%, respectively (Park *et al*, 2002).

The demographic characteristics of cases and controls were compared using the *χ*^2^ test. Conditional logistic regression models were used to calculate odds ratios (ORs) and 95% confidence intervals (CIs). In subgroup analyses stratified by HP IgG antibody, unconditional logistic regression models were used because the matches of cases and controls were not preserved after stratification. ORs were adjusted for smoking history, alcohol consumption, years of education and matching variables. Subgroup analyses stratified by follow-up period (<2.4 years *vs* ⩾2.4 years) were also undertaken. All statistical analyses were performed using SAS v9.1.

## RESULTS

Table 1 shows the baseline characteristics of the study subjects. The mean age of cases was 63 years and two-thirds were male. Of the cases, 34% were never-smokers and 44% never-drinkers and 27% were uneducated. Smoking history and alcohol drinking history, and years of education were not significantly different between cases and controls. In all, 75 cases were non-cardiac gastric cancer and 87 were adenocarcinoma. The median interval from initial blood collection to the diagnosis of gastric cancer was 2.4 years.

Table 2 shows the OR for gastric cancer in relation to HP infection and the virulence factors. HP infection was present in 89% of cases and 90% of controls. Overall, HP infection was not found to be associated with gastric cancer (OR=0.96, 95% CI 0.68–1.36). CagA and VacA seropositivity was not found to elevate the risk of gastric cancer (OR=1.10, 95% CI 0.83–1.47; OR=1.04, 95% CI 0.85–1.28, respectively). The risk of HP infection and CagA and VacA seropositivity were not found to be significantly different for gender (male *vs* female) and the period of follow-up (<2.4 *vs* ⩾2.4 years) (data not shown).

The associations of CagA and VacA seropositivity on the risk for gastric cancer were evaluated stratified by HP IgG antibody (Table 3). Among HP-infected subjects, <10% of them were infected with CagA-negative strains. CagA seropositivity was significantly associated with gastric cancer risk among HP-infected subjects (OR=3.74, 95% CI 1.10–12.73), although not among HP-negative subjects (OR=0.96, CI 0.08–11.66). There was no significant interaction between HP and CagA (p-interaction=0.245). VacA seropositivity insignificantly increased the risk of gastric cancer among HP-infected subjects (OR=1.38, 95% CI 0.81–2.35). However, because there was no subject who was *H. pylori*-negative and VacA-positive, the interaction between *H. pylori* and VacA could not be evaluated.

## DISCUSSION

This nested case–control study suggests that CagA-producing HP increases the risk of gastric cancer in the Korean population. There was no evidence, however, of any significant statistical interaction between HP and CagA. CagA-positive strains have been reported as being more virulent with respect to atrophic gastritis, intestinal metaplasia and gastric cancer development (Hatakeyama, 2004). It may also be relevant that in the Mongolian gerbil, CagA-positive HP strains caused more severe inflammation in gastric mucosa than did CagA-negative strains (Dhar *et al*, 2003).

In a recent meta-analysis of 16 epidemiologic studies, the overall OR for CagA seropositivity among HP-infected subjects was 1.49 (95% CI 1.25–1.77) (Huang *et al*, 2003). Several studies have failed to detect a positive association between VacA protein seropositivity and gastric cancer risk (Shimoyama *et al*, 1999; Yamaoka *et al*, 1999), although Rudi *et al* (1997) reported an elevated risk of gastric cancer (OR=1.74, 95% CI 1.08–2.78) in VacA seropositive participants (Rudi *et al*, 1997).

The risk of gastric cancer associated with CagA observed in the present study was higher than that in the meta-analysis by Huang *et al*. It has been suggested that the distribution and pathogenicities of HP subtypes found in East Asia differ from those found in Western countries (Hatakeyama, 2004). In Europe and the US the *cagA1* subtype of the *cagA* gene is dominant, whereas the *cagA2* subtype, which is more biologically active and virulent, is exclusively found in East Asia (Gonzalez *et al*, 2003). Genotypes for CagA were not investigated in the present study. Nevertheless, if the majority of HP strains infecting the Korean population are the *cagA2* subtype, the higher risk found in the present study than in Western studies concurs with the putative mechanistic role of this subtype in gastric carcinogenesis.

Prospective and community-based cohort design of the present study minimized the possibility of misclassification for exposure. Using the residence registration number, which is a unique identifier for each individual in Korea, the follow-up data linkages were established and enabled complete identification of cancer development status and death.

However, the study also has certain limitations. The small number of gastric cancer patients (*n*=100) and the low frequency of HP-negative subjects (10%) limits the statistical power to evaluate the effect of HP infection and virulence factors. The misclassification of exposure to HP infection due to seroreversion of HP in the elderly with gastric atrophy and the relatively short period of follow-up might have influenced our results (Kikuchi, 2002). But the risks of HP infection and virulence factors were not significantly different for the follow-up period (<2.4 *vs* ⩾2.4 years). The direction of its influence, if it existed, would have been toward the null. Measurement of serum pepsinogen I and II levels would have been helpful in terms of identifying participants with premalignant lesions and preventing the misclassification (Watabe *et al*, 2005).

Our study suggests that CagA-producing HP increases the risk of gastric cancer in the Korean population, although it should be noted that a large proportion of healthy controls are also infected with CagA- or VacA-producing HP. Some nutrients, food components and host genetic polymorphisms may be involved in gastric carcinogenesis associated with HP infection (Hamajima, 2003; Correa, 2004). Further studies on individual genetic susceptibilities and dietary habits, and on the effects of bacterial variants should be pursued.

## Figures and Tables

**Table 1:** Baseline characteristics of the gastric cancer patients and controls

	**Cases (*n*=100) No. (%)**	**Controls (*n*=400) No. (%)**	*P*-value*
*Gender*			
Female	33 (33)	132 (33)	
Male	67 (67)	268 (67)	1.00
			
*Age (year)*			
<65	60 (60)	252 (63)	
⩾65	40 (40)	148 (37)	0.58
			
*Smoking history*			
Never smoker	34 (34)	171 (43)	
Ex-smoker	14 (14)	58 (15)	
Current smoker	52 (52)	171 (43)	0.22
			
*Alcohol consumption*			
Never drinker	44 (44)	179 (45)	
Ex-drinker	12 (12)	33 (8)	
Current drinker	44 (44)	188 (47)	0.49
			
*Education (year)*			
No education	27 (27)	111 (29)	
1–12	71 (71)	283 (71)	
⩾13	2 (2)	6 (2)	0.93
			
*Follow-up duration until diagnosis (year)*			
<2.0	37 (37)		
2.0–4.9	46 (46)		
⩾5.0	17 (17)		
			
*Tumour sites*			
Cardia	7 (7)		
Non-cardia	75 (75)		
Unspecified	18 (18)		
			
*Tumour pathology*			
Adenocarcinoma	87 (87)		
Others	3 (3)		
Unspecified	10 (10)		

* *P*-values were calculated using the *χ*^2^ test.

**Table 2:** Odds ratios and 95% confidence intervals for gastric cancer according to the presence of *H. pylori* IgG antibody and CagA and VacA seropositivity

**Subgroups**	**Cases No. (%)**	**Controls No. (%)**	**aOR^a^ (95% CI^b^)**
*H. pylori*			
Negative	11 (11)	40 (10)	1.0
Positive	89 (89)	360 (90)	0.96 (0.68–1.36)
CagA			
Negative	10 (10)	63 (16)	1.0
Positive	90 (90)	337 (84)	1.10 (0.83–1.47)
VacA			
Negative	36 (36)	158 (40)	1.0
Positive	64 (64)	242 (61)	1.04 (0.85–1.28)

a Odds ratios were adjusted for smoking, alcohol, education using conditional logistic regression model.

b CI: confidence intervals.

**Table 3:** Odds ratios and 95% confidence intervals for gastric cancer according to the CagA and VacA seropositivity stratified by *H. pylori* IgG

**Subgroups**	**Cases No. (%)**	**Controls No. (%)**	**aOR^a^ (95% CI^b^)**
*H. pylori positive*			
CagA			
Negative	3 (3)	35 (10)	1.0
Positive	86 (97)	325 (90)	3.74 (1.10–12.73)
VacA			
Negative	25 (28)	117 (33)	1.0
Positive	64 (72)	243 (68)	1.38 (0.81–2.35)
CagA & VacA			
Others	27 (30)	133 (37)	1.0
Both positive	62 (70)	227 (63)	1.48 (0.88–2.49)
			
*H. pylori negative*			
CagA			
Negative	7 (64)	28 (70)	1.0
Positive	4 (36)	12 (30)	0.96 (0.08–11.66)
VacA			
Negative	11 (100)	40 (100)	
Positive	0 (0)	0 (0)	— —

a Odds ratios adjusted for age, gender, smoking, alcohol, education, area of residence and the year of recruitment using unconditional logistic regression model.

b CI: confidence intervals.
